# How MicroRNA and Transcription Factor Co-regulatory Networks Affect Osteosarcoma Cell Proliferation

**DOI:** 10.1371/journal.pcbi.1003210

**Published:** 2013-08-29

**Authors:** Kathrin Poos, Jan Smida, Michaela Nathrath, Doris Maugg, Daniel Baumhoer, Eberhard Korsching

**Affiliations:** 1 Institute of Bioinformatics, University of Münster, Münster, Germany; 2 Clinical Cooperation Group Osteosarcoma, Helmholtz Zentrum München, German Research Center for Environmental Health, Neuherberg, Germany; 3 Children's Cancer Research Center and Department of Pediatrics, Klinikum rechts der Isar, Technische Universität München, Munich, Germany; 4 Bone Tumor Reference Center at the Institute of Pathology, University Hospital Basel, Basel, Switzerland; Ottawa University, Canada

## Abstract

Osteosarcomas (OS) are complex bone tumors with various genomic alterations. These alterations affect the expression and function of several genes due to drastic changes in the underlying gene regulatory network. However, we know little about critical gene regulators and their functional consequences on the pathogenesis of OS. Therefore, we aimed to determine microRNA and transcription factor (TF) co-regulatory networks in OS cell proliferation. Cell proliferation is an essential part in the pathogenesis of OS and deeper understanding of its regulation might help to identify potential therapeutic targets. Based on expression data of OS cell lines divided according to their proliferative activity, we obtained 12 proliferation-related microRNAs and corresponding target genes. Therewith, microRNA and TF co-regulatory networks were generated and analyzed regarding their structure and functional influence. We identified key co-regulators comprising the microRNAs *miR-9-5p*, *miR-138*, and *miR-214* and the TFs *SP1* and *MYC* in the derived networks. These regulators are implicated in *NFKB*- and *RB1*-signaling and focal adhesion processes based on their common or interacting target genes (e.g., *CDK6*, *CTNNB1*, *E2F4*, *HES1*, *ITGA6*, *NFKB1*, *NOTCH1*, and *SIN3A*). Thus, we proposed a model of OS cell proliferation which is primarily co-regulated through the interactions of the mentioned microRNA and TF combinations. This study illustrates the benefit of systems biological approaches in the analysis of complex diseases. We integrated experimental data with publicly available information to unravel the coordinated (post)-transcriptional control of microRNAs and TFs to identify potential therapeutic targets in OS. The resulting microRNA and TF co-regulatory networks are publicly available for further exploration to generate or evaluate own hypotheses of the pathogenesis of OS (http://www.complex-systems.uni-muenster.de/co_networks.html).

## Author Summary

Osteosarcomas (OS) are bone tumors most frequently affecting children and young adolescents. We do not know much about its molecular pathogenesis hampering personalized therapies to more effectively cure patients. To this day, almost all patients receive adjuvant chemotherapies independent of its necessity. Hence, we need to gain a comprehensive understanding of the molecular pathogenesis of OS to uncover molecular components that can be used as therapeutic targets. MicroRNAs and transcription factors (TFs) are master regulators of the cellular system. They control the amount of genes expressed in a cell at a specific time point that ultimately results in a distinct cellular phenotype. Here, we investigated microRNA and TF co-regulatory networks in OS cell growth as one hallmark in cancer. We uncovered key microRNA and TF regulators that cooperatively control growth-related pathways in OS and proposed potential therapeutic targets. This study illustrates the benefit of analyzing complex diseases from a network perspective because no molecular component functions isolated from the underlying cellular system.

## Introduction

Osteosarcoma (OS) is a rare type of cancer frequently occurring in children and young adolescents [1]. It is a complex tumor typically accompanied by severe genomic instability and extensive mutations hampering the identification of a genetic root [2]–[4]. These genomic alterations affect several genes to a varying extent depending on patient and OS subtype. For instance, frequent mutations and deletions of the tumor suppressor genes *TP53*, *RB1*, and *CDKN2A* and mutations and amplification of the *MYC* locus [5], [6]. However, their interactions in the molecular pathogenesis and the underlying cellular network of OS are poorly characterized.

Recently, attention has been focused on the impact of microRNAs in OS. Besides transcription factors (TFs) that transcriptionally regulate gene expression, microRNAs are a class of small, conserved, non-coding RNA molecules generally acting on the post-transcriptional level. They are mono- or polycistronically transcribed, processed to mature molecules and subsequently incorporated into the RNA Induced Silencing Complex (RISC). Once integrated in RISC, microRNAs are able to select their target genes via binding to partially complementary sequences in the 3′-UTRs of mRNAs that lead to mRNA degradation or translational inhibition. Computational prediction methods revealed that individual microRNAs regulate hundreds of target genes and one target gene might be regulated by several microRNAs [7]. According to Friedman *et al.* [8] around 60% of human genes are predicted to be regulated by multiple microRNAs in a cooperative manner. This huge number of target genes suggests a widespread control of biological processes including differentiation, proliferation, migration, and apoptosis [9].

In cancer, microRNAs might serve as onco- and/or tumor suppressor-microRNAs. Amplification or over-expression of oncogenic microRNAs can down-regulate tumor suppressor proteins. Likewise, deletion or under-expression of tumor suppressor microRNAs might lead to the up-regulation of oncogenes [10]. In addition, more than 50% of microRNA genes are located within fragile sites in the genome and are frequently subjected to chromosomal alterations [11]. In this manner, they can affect cancer development and progression.

MicroRNAs share several regulatory concepts with TFs, e.g. they simultaneously regulate many target genes and cooperate with other regulators. However, TFs activate or repress their target gene expression whereas microRNAs regulate their targets primarily through repression to fine-tune cell-specific gene regulatory programs [12]. Because the expression of microRNAs often depends on TF regulation and vice versa, it is not surprising that both families of regulators are tightly related to each other in gene regulatory networks. The coordinated transcriptional regulation of microRNAs and their target genes by TFs is a recurrent network motif. The two types of gene regulators frequently form 3-node feedforward loops (FFLs) with common target genes [13]. Recently, Sun *et al.* [14] extended this regulatory motif to 4-node FFLs by integrating additional TF target genes. The extension of 3-node to 4-node motifs illustrated a more detailed model of the oncogenesis of glioblastoma by recruiting additional disease genes not directly targeted by microRNAs.

Several studies have shown an involvement of microRNAs in the pathogenesis of OS. They demonstrated down-regulation of *miR-143* in OS progression [15], up-regulation of the oncogenic *miR-17∼92* cluster in OS cells [16], and regulatory functions for *miR-199a-3p* [17], *miR-21* [18], and *miR-125b* [19] in OS cell proliferation and migration. Additional genome-wide microRNA analyses suggested sets of microRNAs to discriminate OS from osteoblasts and bone tissue [20]–[23]. All studies proposed the use of microRNAs as biomarkers in OS that might correlate with clinico-pathological parameters. However, those studies lack a comprehensive analysis of the functional consequences of aberrant microRNA expression in OS. Analyzing microRNAs in the context of their microRNA and TF co-regulatory networks might provide a deeper understanding of the pathogenesis of OS.

**Figure 1:**
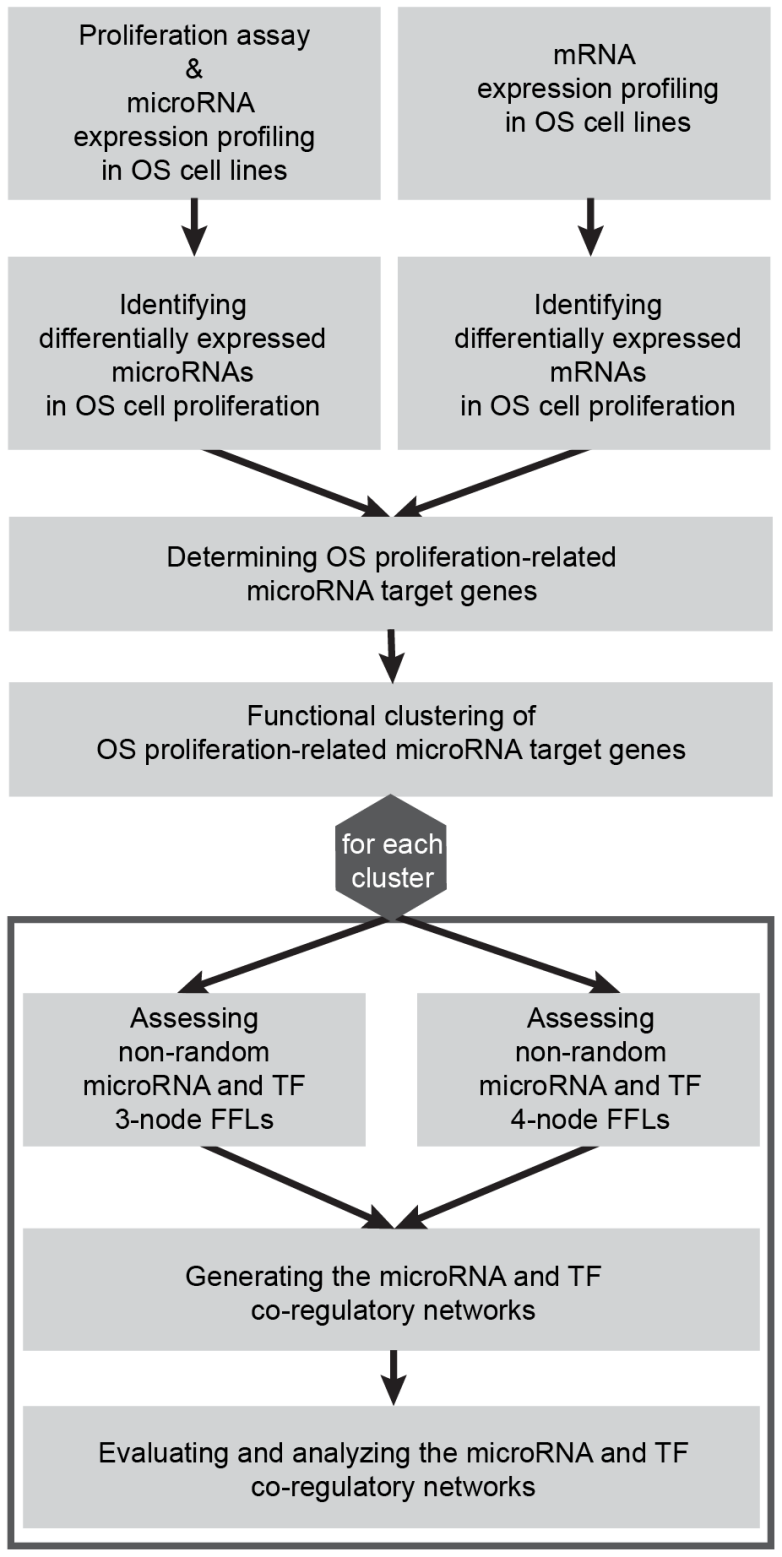
Workflow. The figure illustrates the procedure of the present study determining microRNA and TF co-regulation in OS cell proliferation.

In this study, we joined different data sources to analyze the contribution of microRNA and TF co-regulatory 3-node and 4-node motifs to the proliferative activity of OS cells. First, we divided seven OS cell lines into high and low proliferation groups by performing proliferation assays. Expression analysis based on these groups yielded differentially expressed (DE) microRNAs and mRNAs. Second, high efficacy microRNA target genes were obtained by integrating computational predicted targets with DE mRNAs. Only microRNAs with significantly enriched target genes were considered in the analysis. Third, microRNA target genes were clustered according to their functional similarity to explore their distinct biological processes. Fourth, transcription factor binding site (TFBS) information was added to assemble 3-node and 4-node motifs of non-random microRNA and TF co-regulator pairs. Fifth, the resulting 3-node and 4-node motifs were merged to form microRNA and TF co-regulatory networks to examine the coordinated regulation of microRNAs and TFs (Figure 1).

Here, we present the first study analyzing microRNA and TF co-regulation in OS and uncover critical microRNA players of the functional processes implicated in OS cell proliferation.

## Results

### OS cell proliferation and differential microRNA expression

**Figure 2:**
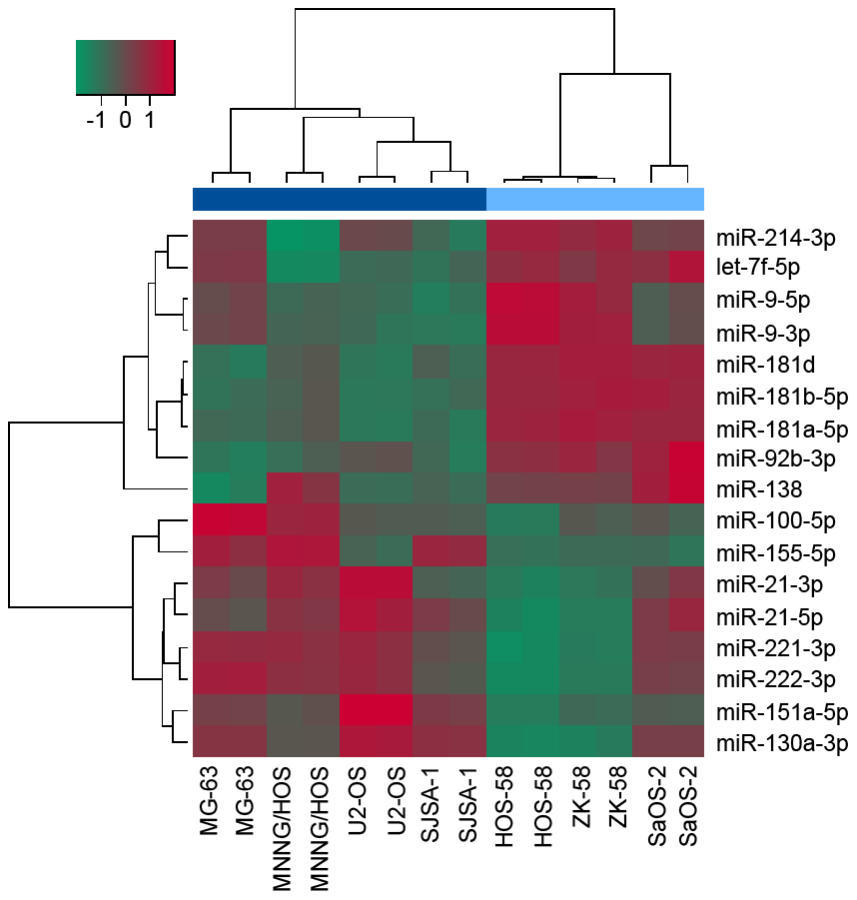
Clustering of differentially expressed microRNAs. The heatmap illustrates the expression profiles of DE microRNAs (log2 FC≥|1| & FDR<0.05, y-axis) among all OS cell samples (x-axis). High (dark blue) and low (light blue) proliferative OS samples are clearly separated. The red/green color code corresponds to the expression deviation from the average expression among all samples. Complete-linkage clustering was performed with the Pearson correlation as distance metric.

In order to investigate the deregulated microRNA and TF co-regulatory networks of proliferative OS cells, we used seven authenticated OS cell lines. The cell lines were divided according to their proliferative activity by performing a proliferation assay. Four OS cell lines exhibited a high proliferative activity with a doubling time <10 hours while three showed less proliferation (Table 1). MNNG/HOS, U2-OS, and SJSA-1 showed additional extensive migratory capabilities. The expression analysis of the microRNAs was based on these two proliferation groups. The analysis yielded nine down-regulated and eight up-regulated microRNAs that passed the differential expression criteria (False discovery rate (FDR) <0.05 & log2 Fold change (FC)≥|1|, Table 2). The derived DE microRNAs have been reported in association with neoplastic disease either due to oncogenic or tumor suppressor properties. Hierarchical clustering of them clearly separated the OS cell samples according to their proliferative activity (Figure 2). Hence, we selected the DE microRNAs as candidates that might affect OS cell proliferation for further analysis.

**Table 1:** Proliferation and migration potential of OS cell lines.

Cell line	Proliferation	Migration
HOS-58	low	low
SaOS-2	low	low
ZK-58	low	low
MG-63	high	low
MNNG/HOS	high	high
SJSA-1	high	high
U2-OS	high	high

**Table 2:** Differentially expressed microRNAs dependent on OS cell proliferation.

microRNA	log2 FC	FDR	References
miR-181a	−2.28	0.000013	[20]
miR-9-3p	−2.01	0.00443	[23]
miR-181d	−1.82	0.000028	[23]
miR-138	−1.81	0.03828	[33]
miR-214	−1.59	0.03083	[20]
miR-9-5p	−1.44	0.00525	[23]
miR-181b	−1.17	0.000028	[20]
let-7f	−1.09	0.00211	[73]
miR-92b	−1.06	0.000149	[74]
miR-130a	2.77	0.00698	[75]
miR-21-5p	2.31	0.03760	[18]
miR-155	2.05	0.00382	[76]
miR-222	1.87	0.00698	[77]
miR-221	1.45	0.00823	[77]
miR-100	1.26	0.03827	[22]
miR-21-3p	1.23	0.03760	[23]
miR-151-5p	1.00	0.00698	[78]

DE microRNAs are shown with their corresponding log2 FC and FDR. A negative log2 FC indicates that microRNAs are down-regulated in high proliferative OS cells and vice versa. The references demonstrate an implication in cancer of the respective microRNA.

### OS proliferation-related microRNA target genes

To explore the functional consequences of DE microRNAs on OS cell proliferation, we determined their target genes by integrating gene expression profiles with computational predicted target genes.

First, the expression analysis of mRNAs resulted in a total of 666 up-regulated and 610 down-regulated mRNAs. We applied loose filter criteria for DE mRNAs without correcting for multiple tests (p-value<0.05 & log2 FC≥|0.7|) because microRNA regulation might lead to subtle changes in gene expression. Next, we superimposed the DE genes with predicted microRNA targets to obtain target genes affecting OS cell proliferation. We assumed that microRNAs exhibit an inverse regulatory relationship to their functional target genes, i.e. microRNA expression is inversely correlated to its target gene expression. Hence, down-regulated targets were assigned to up-regulated microRNAs in high proliferative OS cells and vice versa.

**Figure 3:**
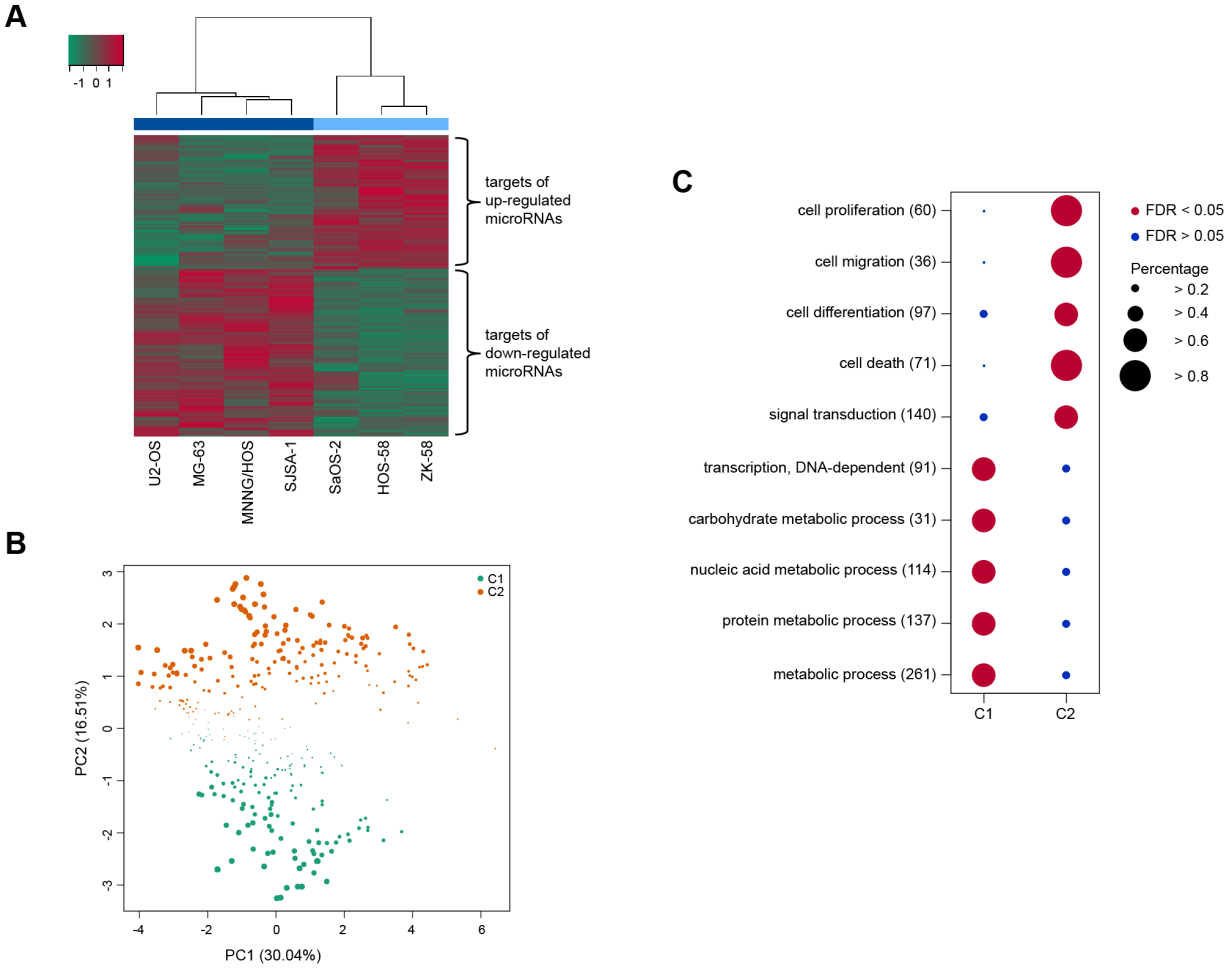
Functional implications of microRNA target genes in OS proliferation. (**A**) Differentially expressed microRNA target genes. The heatmap shows the expression of DE microRNA target genes (log2 FC≥|0.7| & p-value<0.05, y-axis) among all OS cell samples (x-axis). High (dark blue) and low (light blue) proliferative OS samples are grouped into distinct clusters. The red/green color code corresponds to the expression deviation from the average expression among all samples. Complete-linkage clustering was applied with the Pearson correlation as distance metric. (**B**) Principal component analysis based on GO functional similarity. The figure draws the first (x-axis) and second (y-axis) principal components based on the GO functional similarity of microRNA target genes. The target genes are color coded according to their cluster membership determined by FCM. The radius of each gene represents the strength of association to its cluster. (**C**) Comparison of the GO enrichment analyses of the distinct clusters. Each row corresponds to a specific biological process category. The number of genes included in each category is indicated in the round brackets. Circle sizes correspond to the percentage of row-genes located in a cluster. Significantly enriched categories are color coded in red and random categories in blue.

To exclude DE microRNAs with random association to OS cell proliferation, we tested for microRNA target gene enrichment within the list of DE genes. Among the 17 DE microRNAs, 12 are significantly enriched due to their targets (FDR<0.05, Table S1). To account for different numbers of targets that might influence the enrichment analysis, we also computed the target gene enrichment of 1,000 permuted samples. The permutation procedure confirmed previous results (Figure S1). Consequently, we excluded 5 microRNAs from further analyses (*miR-92b*, *let-7f*, *miR-9-3p*, *miR-151-5p*, and *miR-100*). The remaining 12 OS proliferation-related microRNAs are implicated in the regulation of 474 target genes. Hierarchical clustering of the target genes resulted in a distinct separation of the high and low proliferative OS cell samples (Figure 3A).

### Functional clustering of microRNA targets

We further investigated the underlying biological processes of OS proliferation-related microRNAs. We classified microRNA target genes according to their functional similarities of their gene ontology (GO) biological process terms using fuzzy c-means clustering (FCM). After determining FCM parameters (Figure S2), we obtained two clusters. Principal Component Analysis (PCA) supported the results. The first two components separate the determined clusters (Figure 3B). Cluster C1 consists of 172 members and cluster C2 contains 212 members. The remaining 90 microRNA targets could not be annotated with a GO biological process term and were excluded from further analysis. The clustering suggested that the microRNA targets can be classified into two broad functional classes.

GO enrichment analyses revealed that members of C1 are mainly involved in metabolic processes like protein modification, nucleic acid metabolism, and carbohydrate metabolism, whereas members of C2 are implicated in signal transduction pathways leading to proliferation, differentiation, apoptosis, and migration. Both clusters demonstrate that cancer cells adapt metabolic processes for cell proliferation and survival [24]. A comparison between the five most informative GO terms (FDR<0.05) illustrating the specific biological aspects of each cluster is shown in Figure 3C.

### Identifying microRNA and TF co-regulated target genes

Transcriptional regulation of TFs is tightly coupled with the post-transcriptional regulation of microRNAs. We utilized their 3-node and 4-node co-regulatory motifs to study DE microRNA and TF co-regulation in OS cell proliferation for each functional cluster.

**Figure 4:**
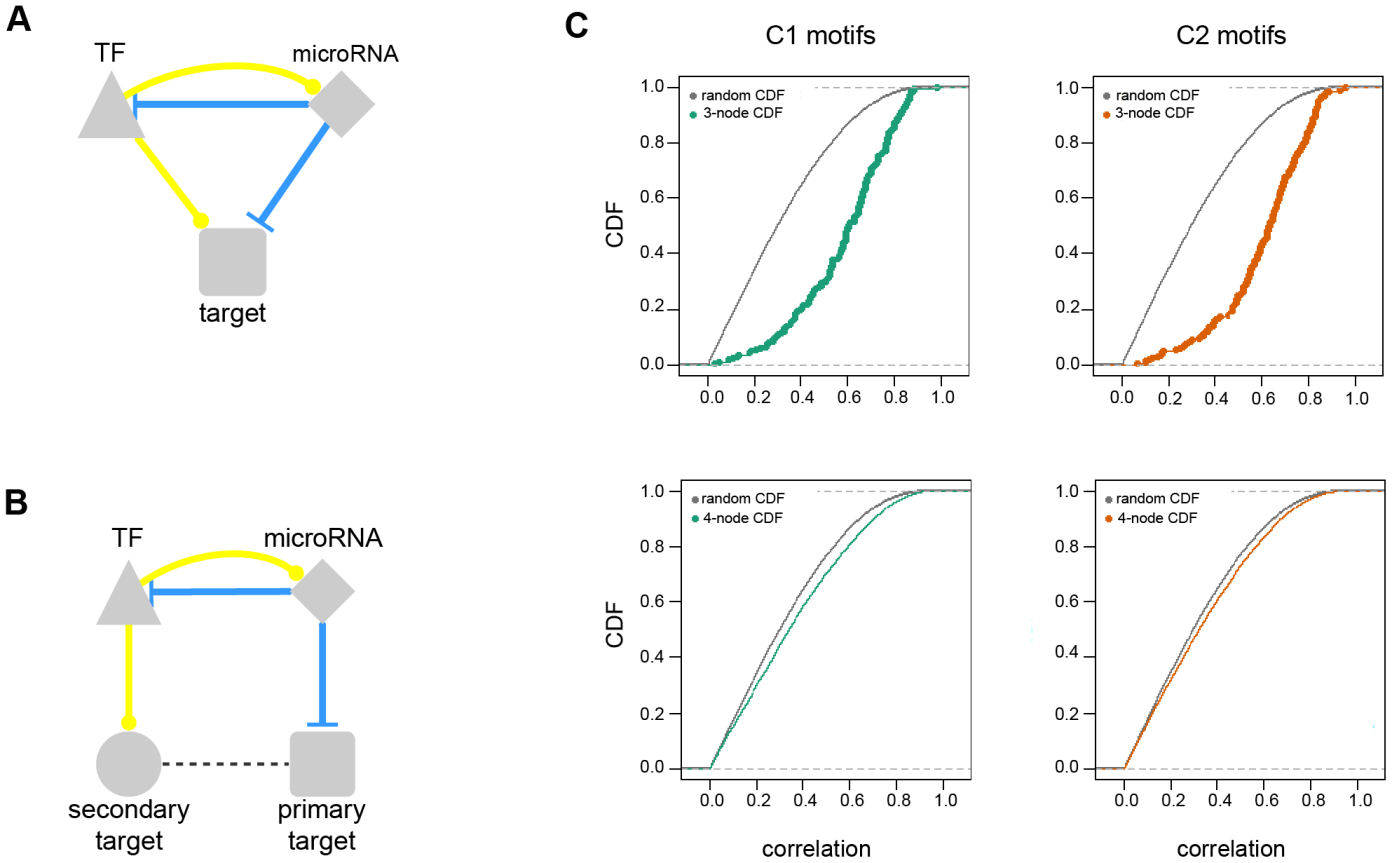
MicroRNA and TF co-regulatory motifs. Schematic illustration of (**A**) 3-node and (**B**) 4-node FFL motifs. A 3-node motif contains a microRNA, a TF, and a commonly regulated target gene. In contrast, 4-node FFLs comprise a microRNA, a TF, a microRNA target gene (primary target), and a TF target gene (secondary target) that interacts with the primary target. MicroRNAs are indicated with diamonds, TF with triangles, primary targets with rectangles, and secondary targets with circles. (**C**) Coexpression of microRNA and TF target genes. The plot shows the observed and random cumulative distribution functions (CDFs, y-axis) of the Pearson correlation coefficients (x-axis) between any gene pair regulated by a specific microRNA and TF 3-node and 4-node motif. The observed and random CDFs are compared using the KS test. The p-values are indicated within the plots. Green and orange color codes correspond to the distinct clusters C1 and C2.

Every possible 3-node and 4-node FFL motif was determined to assess significant microRNA and TF combinations (FDR<0.2) by using the hypergeometric test (Table S2 and S3). For the 3-node FFL, we obtained non-random microRNA and TF pairs with common target genes (Figure 4A). For 4-node FFL motifs, we assessed non-random microRNA and TF pairs that regulate gene neighbors in the protein interaction network (Figure 4B). The individual 3-node and 4-node FFL motifs are listed in Table S4 and S5, respectively.

**Figure 5:**
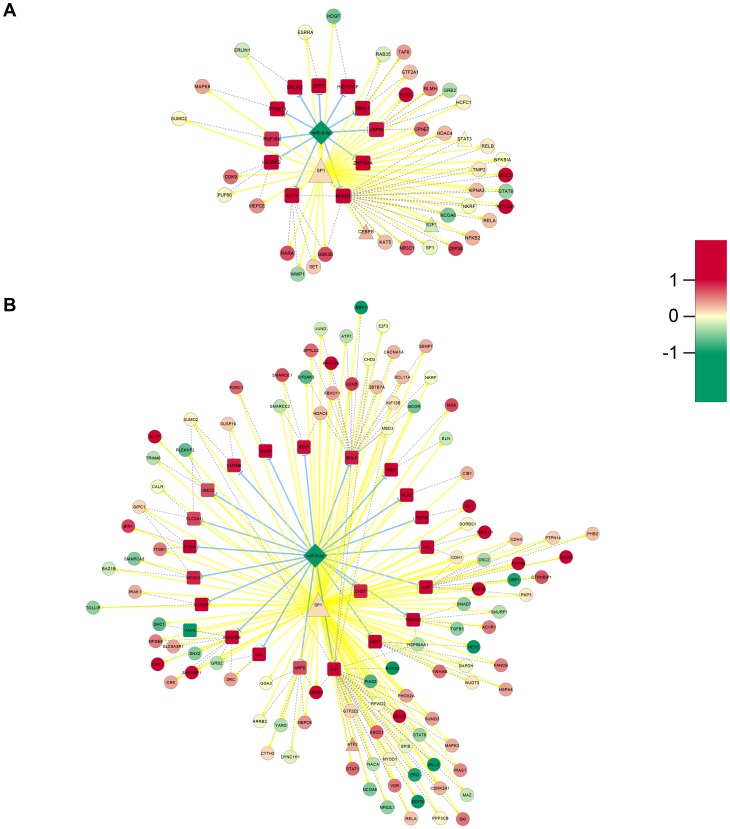
*miR-9-5p* and *SP1* co-regulatory motifs. The co-regulatory motifs of *miR-9-5p* and *SP1* are illustrated as graphs with nodes and edges for (**A**) C1 and (**B**) C2. MicroRNAs are marked with diamonds, TFs with triangles, primary targets with rectangle, and secondary targets with a circle. Yellow edges tag protein-DNA interactions, blue edges microRNA-target interactions, and dashed edges protein interactions. The red/green color code presents the corresponding log2 FC.

Subsequently, we analyzed the co-regulated target genes of significant microRNA and TF combinations. The results are summarized in Table 3. Noticeably, the microRNA and TF duo with the highest number of co-regulated target genes in both functional clusters is *miR-9-5p* and *SP1* (Figure 5) indicating a prominent role in OS cell proliferation.

**Table 3:** Summary of proliferation-related microRNA and TF co-regulations.

Cluster	Motif	microRNA	TF	Primary targets	Secondary targets	microRNA and TF pairs
**C1**	3-node FFL	10	17	58	—	29
	4-node FFL	8	20	69	227	23
	total	10	29	85	213	46
**C2**	3-node FFL	10	31	88	—	58
	4-node FFL	9	21	81	581	29
	total	10	35	117	550	70

The table specifies the number of common and interacting target genes between non-random microRNA and TF pairs for 3-node and 4-node motifs and for the total co-regulatory network they form.

Further, we examined the co-expression of genes co-regulated by the same microRNA and TF pairs. We computed the Pearson correlation coefficients between co-regulated gene pairs as a measure of their co-expression. The distribution of the resulting correlation values was compared to the correlation distribution of random genes by the Kolmogorov-Smirnov (KS) test. The co-expression of co-regulated gene pairs tends to be significantly higher than for random genes (p-value<2.2×10^−16^, Figure 4C). This result supports the hypothesis of non-random microRNA and TF co-regulation within the list of their common or interacting target genes and suggests a similar functional context for their targets.

### Generating microRNA and TF co-regulatory networks

Subsequently, we constructed the microRNA and TF co-regulatory networks that highlighted the combinatorial regulation patterns and regulated biological processes of microRNAs and TFs. The networks of C1 and C2 were generated by joining all significant co-regulatory relationships of microRNAs and TFs (Table 3). The resultant microRNA and TF co-regulatory networks are provided for full exploration on our website (http://www.complex-systems.uni-muenster.de/co_networks.html).

To assess the contribution of individual nodes in the co-regulatory networks on the networks' stability and robustness, we calculated the node degree and betweenness centrality parameters. The node degree distributions are highly right skewed. A large fraction of nodes shows a low degree and only few nodes have high degrees (Figure S3). Almost all microRNAs and TFs are located at higher node degrees as indicated by their average node degrees (C1: microRNAs 19 and TFs 19, C2: microRNAs 25 and TFs 49). We expected that finding as microRNA and TF co-regulation is the main subject of the present study.

Each network contains three types of nodes, namely microRNAs, TFs, and target genes. We ranked the nodes according to their node degrees and node type. The top 25% of microRNAs and TFs and the top 5% of target genes were considered as hubs in the C1 and C2 networks (Table S6). We detected the hub microRNA *miR-214* and the hub TFs *CREB1*, *SP1*, and *ZIC2* in both networks suggesting a central function in OS cell proliferation. Strikingly, around 50% of microRNA and TF target gene hubs in the two networks are TFs themselves. The microRNA and TF co-regulatory network derived from C1 contains *ATF6*, *GTF2A1, HIVEP2, KLF5*, *LMO3*, *NFKB1*, and *TBPL1* and the C2 network comprises *BCL6, BCL6B, E2F4, HES1, JUN, LMO4, RARA, REST, SIN3A, TCF7L2*, and *ZBTB16*. Some of these TFs (*ATF6, E2F4, JUN, RARA*, and *REST*) are implicated in building 3-node and 4-node FFL motifs in one or two networks. The remaining TFs were either not existent in the UCSC conserved TFBS track or do not produce any significant FFL. Additionally, we found epigenetic modulators and genes involved in protein modification processes, like protein ubiquitination and phosphorylation. As already mentioned, the microRNA targets in C2 are associated with signal transduction for maintaining OS cell proliferation. Among the hub genes in the network derived from C2, 30% of target gene hubs (*AMOT*, *ARF6, CACNA1A, CTNNB1*, *GRB2, NOTCH1*, *PDGFRB*, *PIK3R1*, *SMAD7*, and *TGFBR2*) are related to signaling pathways that participate in cell proliferation, survival, and migration.

Moreover, we assessed over-represented functional pathways derived from the KEGG database [25]. The enriched categories (FDR<0.05) are shown in Table S7. We detected an enrichment of genes involved in the cell cycle and cancer related pathways in both networks. We expected to observe these functional categories as we analyzed the proliferative potential of OS tumor cell lines. Further, we observed a similar functional trend between the C1 and C2 networks as for their corresponding functional clusters. Within the co-regulatory network of C2, signaling pathways are significantly over-represented such as the *MAPK*-, *TGFB*-, and *WNT*-signaling pathways. In contrast, the network of C1 comprises a significant number of genes required for the basal transcription machinery.

### Tightly connected microRNA and TF co-regulated subnetworks

After examining the global co-regulation patterns of microRNAs and TFs in both networks, we were interested in sets of microRNAs and TFs that co-regulate densely connected network modules. To investigate the local structure of the OS proliferation-related co-regulatory networks, the walktrap algorithm was applied [26].

The algorithm obtained six modules within the metabolic network of C1 (Figure S4) and six modules in the signaling network of C2 (Figure S5). The size and node types within each module are indicated in Table 4. Strikingly, *miR-9-5p* is located in the largest module and is regulated through the TFs *ATF2, BACH1*, *CREB1*, and *SP1* in both networks. As mentioned before, *miR-9-5p* and *SP1* co-regulate the largest number of target genes and thus indicate a prominent function in OS cell proliferation.

**Table 4:** MicroRNA and TF co-regulatory subnetworks.

ID	microRNA	TF	nodes	edges	associated biology	p-value^a^
C1.1	miR-221, -222, -130a, -21-5p	JUN, HSF2, SRF, HSF1, FOXC1, IRF7, FOXO1, YY1	72	156	transcription	2.0×10^−03^
C1.2	miR-21-3p	E2F1, STAT3	33	53	phosphorylation	3.0×10^−03^
C1.3	miR-214	BPTF, ZEB1, E2F4, NFIL3	62	120	cell cycle	5.6×10^−03^
C1.4	miR-9-5p	CREB1, CEBPB, ATF2, SP1, ZIC2, TCF3, BACH1	91	248	transcription	1.0×10^−11^
C1.5	miR-181a, -181b	MEF2A, TBP, SREBF1, MAX, POU2F1	60	142	chromosome organization	8.1×10^−04^
C1.6	miR-138	MYC, KLF12	19	32	—	—
C2.1	miR-138	MYC, FOXC1, USF1, ZIC1, FOXJ2	157	377	transcription	1.0×10^−17^
C2.2	miR-21-5p, -21-3p	STAT3, IRF9, SOX9, MEF2A	70	124	transport	5.6×10^−03^
C2.3	miR-222, -221, -214	RORA, NR3C1, FOXO1	127	235	transcription	1.0×10^−04^
C2.4	miR-130a	CEBPB, ZEB1, GATA2, SRF, YY1	87	144	differentiation	9.8×10^−04^
C2.5	miR-181a, -181b	POU2F1, KLF12, NFIL3	21	41	—	—
C2.6	miR-9-5p	CREB1, TP53, NR2F2, NFIC, EGR1, ATF6, ATF2, PAX6, SREBF1, SP1, BACH1, NFATC1	247	887	transcription	4.1×10^−12^

For each module the comprised microRNAs, TFs, number of nodes and edges, and associated biological aspects with corresponding p-values are presented.

a ES≥2.0.

— no significant associated biological aspect.

Further, we run the Functional Annotation clustering Tool of the DAVID database [27] to classify the distinct network modules according to their GO biological process and molecular function terms. We annotated each module with the biological aspect of its maximum enrichment score (ES). Among the 12 modules, five are mainly involved in transcriptional regulation processes, which is in accordance with previous studies that illustrated that microRNAs function via TFs to regulate various biological processes like cell proliferation [28], [29]. Despite the top scored biological associations, one module (C2.1) is related to negative regulation of differentiation (ES>3.9), particularly to osteoblast differentiation due to the genes *CDK6*, *MEN1*, *SKI*, *SMAD3*, and *SOX2*, which might provide a link to the pathogenesis of OS (Figure S5A). Within this module, the TF *MYC* (node degree 83) co-regulates several targets with *miR-138* (node degree 25). The top ranked target gene in this module is *SIN3A* (node degree 57).

## Discussion

OS is a complex tumor with varying degrees of genomic alterations and affected disease genes. This genomic complexity makes it difficult to identify a genetic cause and deregulated pathways in the pathogenesis of OS. Systems biological approaches provide tools to investigate the interactions between candidate genes by integrating different data sources on the network level. Thereof, cooperative or compensative effects between candidate genes might be observed. Hence, network-based approaches seemed to be ideally suited to study the implication of functional pathways in OS. This study represents the first attempt to investigate microRNA and TF co-regulatory networks in the pathogenesis of OS. In the course of the study, several data sources were integrated, namely microRNA and mRNA expression profiles, TFBS information, and protein interaction data. Therewith, we aimed to unravel possible candidate genes and their interplay that ultimately result in a high proliferative phenotype of OS cell lines. The derived microRNA and TF networks are publicly available (http://www.complex-systems.uni-muenster.de/co_networks.html).

### OS proliferation-related microRNAs are associated with a proliferative phenotype

The microRNA and TF co-regulatory networks modeling OS cell proliferation are based on 12 proliferation-related microRNAs. Among these microRNAs, 11 were previously mentioned in OS [20], [22], [23], [18], whereas *miR-138* was exclusively obtained in this study. Previous studies focused on global microRNA alterations in OS with respect to osteoblast cells and bone tissue. However, microRNA expression was partially inconsistent between different studies. Namløs *et al.* [23] hypothesized that contradictory microRNA regulation in different genome-wide studies might be explained due to distinct differentiation stages of OS progenitor cells. In this study, the DE microRNAs were assessed between high and low proliferative OS cells. The varying proliferation activity between these cell lines might reflect distinct differentiation stages of their progenitor cells. This might explain the detection of proliferation-related microRNAs in global OS studies.

In different biological contexts the down-regulated microRNAs have been associated with decreased proliferation [30]–[33]. In turn, up-regulated microRNAs have been related to an increased proliferation potential [34]–[37]. These studies emphasize the proposed influence of the derived microRNAs on OS cell proliferation.

### *miR-9-5p* and *SP1* co-regulated targets are highly involved in OS cell proliferation

The assembly of non-random microRNA and TF co-regulators revealed several interesting combinations. Based on the huge number of co-regulated target genes, the most notably co-regulation duo is the *miR-9-5p* and *SP1* pair. Depending on the derived cluster, these co-regulators seem to affect distinct functional pathways due to their target genes.

The C1 derived *miR-9-5p* and *SP1* co-regulated target genes seem to participate in *NFKB*-signaling. Commonly regulated target genes include the TFs *NFKB1*, *NFKB2*, *RELA*, *RELB*, and *BCL3* and the inhibitors *NKRF*, *NFKBIA*, and *TNIP2* that cooperatively activate or block target gene expression of *NFKB*, respectively [38]. This pathway is implicated in OS cell proliferation [39], and *NFKB1* is an experimentally validated target gene of *miR-9-5p* [40]. Furthermore, a regulatory circuit including *SP1/NFKB1/HDAC* and *miR-29b* is known to induce leukemic growth [41]. Thus, *miR-9-5p* might function in a similar context in OS than *miR-29b* in leukemia.

On the other hand, the C2 derived *miR-9-5p* and *SP1* co-regulated genes might be involved in focal adhesion. We observed cadherins (*CDH1*, *CDH2*, *DSC2*, and *DSG2*), further cell adhesion molecules (*e.g. FN1*, *ITGA6*, *ITGB1*, *JUP*, *PKP3*, and *VCL*), and calcium signaling receptors (e.g. *CACNA1A* and *CALR*). According to StringDB [42], almost all commonly regulated genes interact with each other indicating a functional association (Figure S6). A possible pathway of *miR-9-5p* and *SP1* co-regulation could implicate *CDH1* and *CTNNB1* that modulate cell proliferation [43]. *CTNNB1* is not a commonly regulated target gene of *miR-9-5p* and *SP1*. However, we observed some of its binding partners (e.g. *CDH1*, *CDH3*, *CTNNBPI1*, *RUNX2*, and *SMAD7*). Additionally, *CTNNB1* is a hub gene in the C2 co-regulatory network.

Moreover, increased expression of *miR-9-5p* resulted in down-regulation of the *NFKB-SNAIL* pathway and simultaneously to up-regulation of *CDH1* in melanoma cells [44]. All these observations suggest a central role of *miR-9-5p* and *SP1* co-regulation in OS cell proliferation.

### Functional implications of the *miR-138*-*MYC*-*SIN3A* module in the pathogenesis of OS

Merging significant microRNA and TF co-regulators resulted in co-regulatory networks of C1 and C2. The networks provided a global view on microRNA and TF co-regulation in OS cell proliferation. To analyze the local co-regulation patterns within the networks, we specifically extracted densely connected modules. One of these modules, namely the C2.1 module, is implicated in negative regulation of differentiation, particularly osteoblast differentiation. It contains the hubs *miR-138*, *MYC*, and *SIN3A*. We hypothesize a function for members of C2.1 that might be specific for the pathogenesis of OS. According to StringDB [42], the genes in the module form a densely connected network illustrating a tight functional relationship (Figure S7). The module comprises members of the cell cycle (*CCND1, CCND3*, *CDKN1A, CDKN2C*, and *CDK6*), all involved in the *RB1*-pathway [45]. The complex of the module members *SIN3A*, *NCOR1*, *SKI*, and *HDAC* can bind to *RB1* and repress *E2F* target genes [46]. Therefrom, we assumed a connection between module members and *RB1*, which has been reported to be frequently deregulated in OS [5].

Further, *SIN3A* is an experimentally validated target gene *of miR-138* [47]. In the global co-regulatory network of C2 as well as in C2.1 module, it depicts a hub gene. Its role in cancer is contradictory, on the one hand it shows tumor suppressor functions [48]. On the other hand, it acts in tumor growth [49]. Taken together with the fact that *SIN3A* can interact with *RB1*, we suggest a possible role for *SIN3A* in the pathogenesis of OS.

### Model of OS cell proliferation

**Figure 6:**
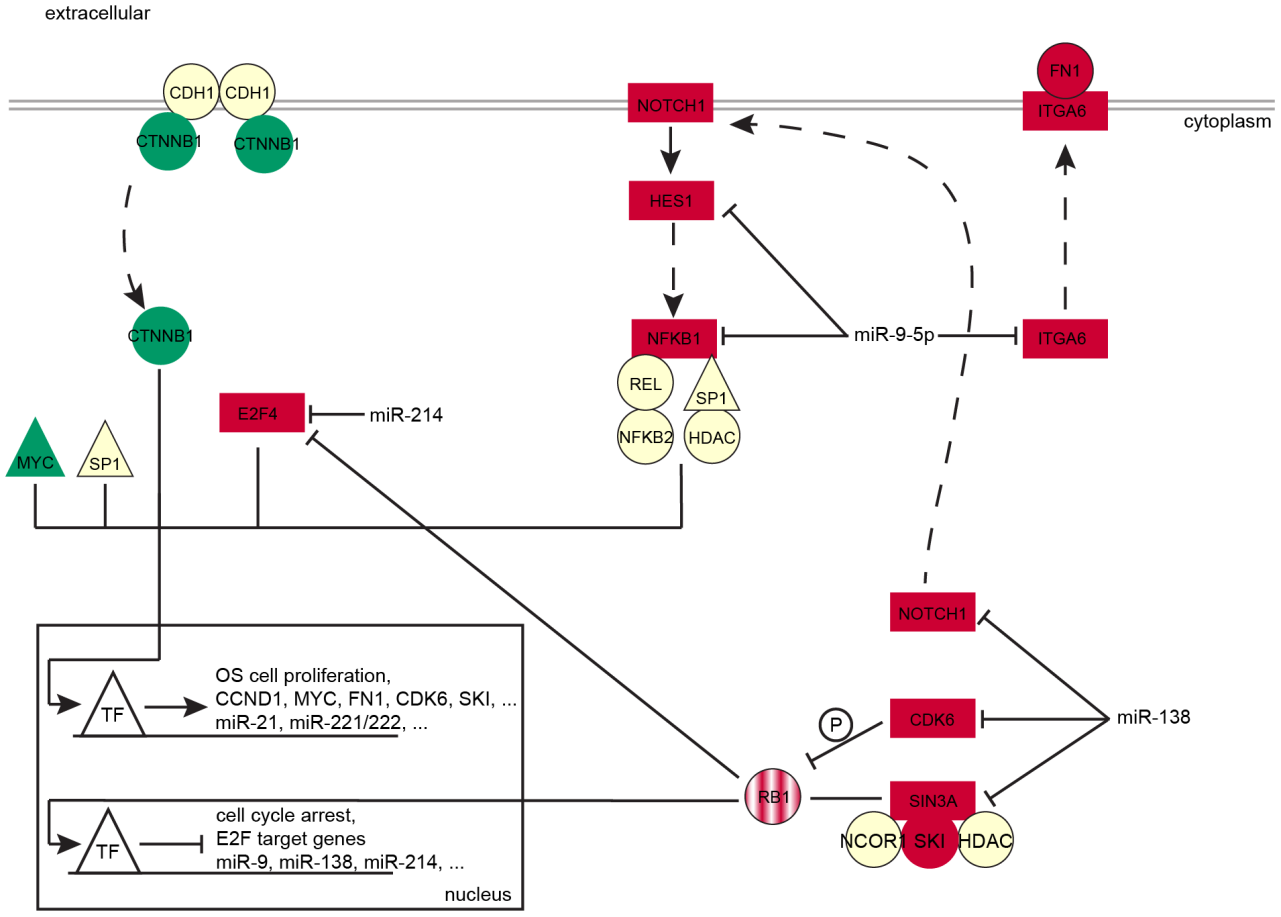
Model of OS cell proliferation. The picture illustrates the proposed model of OS cell proliferation co-regulated by *miR-9-5p*, *miR-138*, and *miR-214* and the TFs *SP1* and *MYC*. These regulators control mainly components of the *NFKB*- and *RB1*-pathway and of focal adhesion processes. Primary microRNA targets are rectangular and secondary targets are ellipse shaped. Red marks up-regulated genes in proliferative active OS cell lines and green indicates down-regulated genes. White nodes represent genes not DE in OS cell proliferation. Solid and dashed arrows illustrate direct and indirect functional associations, respectively. *RB1* is shaded in red to indicate that it is no member of the global networks.

After examining and discussing the structural and functional aspects of the co-regulatory networks, we integrated the main results of the present study into a potential model of OS cell proliferation (Figure 6). The focus of the model is on microRNA and TF co-regulation of the microRNAs *miR-9-5p*, *miR-138*, and *miR-214* and the TFs *SP1* and *MYC*.

In proliferative active OS cell lines, *miR-9-5p*, *miR-138*, and *miR-214* are significantly down-regulated leading to the up-regulation of their direct target genes *CDK6*, *E2F4*, *HES1*, *ITGA6*, *NFKB1*, *NOTCH1*, and *SIN3A*. *CDK6* phosphorylates *RB1* and therewith the *RB1*/*SIN3A*/*SKI*/*NCOR1*/*HDAC* complex cannot repress *E2F4* target gene expression [45], [46]. Activated *NOTCH1* induces *HES1* and sustains *NFKB*-signaling through *NFKB1*, *NFKB2*, *RELA*, *RELB* [50]. *CTNNB1* stabilizes cell-cell adhesions in complex with *CDH1*. Unbound *CTNNB1* can translocate to the nucleus [43]. All these signals end up in the nucleus where they induce expression of proliferation promoting genes like microRNAs up-regulated in high proliferative OS cells, CCND1, and FN1. In turn, they repress genes implicated in growth-arrest.

### Conclusion

The resulting microRNA and TF co-regulatory networks display a detailed picture of the regulation of OS cell proliferation. In the present study, we concentrated on distinct functional aspects unraveled from the networks. The outcome suggests that down-regulation of *miR-9-5p*, *miR-138*, and *miR-214* results in a strong proliferative phenotype of OS cells due to their impact on *NFKB*- and *RB1*-signaling and on focal adhesion.

Our study provides potential therapeutic targets in OS and proposes concepts for further research. In addition, we demonstrated how systems biological approaches support the analyses of complex diseases.

## Materials and Methods

### Data sets

We used microRNA and mRNA expression data of seven authenticated OS cell lines, six from our previously published study [16] and one additional (ZK-58). Prior to microarray analyses, RNA was isolated and further processed as described in [16]. MicroRNA and mRNA expression profiles were determined on Exiqon's miRCURY LNA and Affymetrix's Human Gene 1ST arrays, respectively.

Conserved TFBSs (hg19) were downloaded from the UCSC Table Browser [51]. The track contained predicted TFBSs conserved in the human/mouse/rat alignment that were determined by using the Transfac Matrix Database 7.0 [52]. Protein interaction data were obtained from BioGRID release 3.1.92 [53].

### Proliferation assay to group OS cell lines

The OS cell lines were evaluated regarding their proliferative, migrative, and invasive potential by using in vitro-assays (BD Biosciences). Cells utilized in the assays showed 60 to 80% confluence growth. Prior to the assays cell lines were synchronized to ensure a uniform cell growth.

To analyze OS cell proliferation, duplicates of each cell line (1×10^5^ cells) were seeded in 25 cm^2^ cell culture flasks. Cells were harvested at 24, 48, 72, 96, and 168 hours of growth. The cell number was determined by an automated cell counter (Beckman Coulter). For each cell line and time point, the mean cell number was calculated to estimate the growth rate and subsequently the doubling time.

Further, the Biocoat Matrigel Invasion Assay and a migration assay (BD Biosciences) were performed for each cell line in duplicate with matrigel-coated and uncoated inserts, respectively. Experiments were performed according to the manufacturer's instructions. Evaluation of invaded and migrated cells was done after 24 and 48 hours by light-microscopic analysis. Ten visual fields (magnification 10×) were analyzed by counting stained cells on the membranes.

### Expression analysis of microRNAs and mRNAs

The expression data sets were analyzed using the Bioconductor package limma [54]. DE genes between high and low proliferative OS cells were determined using eBayes [54].

MicroRNA expression data were annotated with miRBase release 18 [55], background corrected by normexp+offset 10 [56], and normalized with printtiploess followed by RQuant [57]. In the differential expression analysis we considered the top 75% of microRNA probes that showed largest variation over all samples. Multiple test correction was performed using Benjamini and Hochberg's FDR approach [58].

The mRNA expression data were preprocessed by the Bioconductor package affy [59]. The Affymetrix Human Gene 1 ST array contains probes mapping among the whole transcript. Therefore, we filtered probes that mapped to exons present in at least 80% of a gene's transcripts to get one stable expression value per gene. Transcripts were derived from Ensembl release 63 [60]. The raw probe intensities were background corrected, normalized, and summarized to the gene-level by applying the robust multi-array average algorithm (rma) [59].

### MicroRNA and TF target prediction

Predicted microRNA targets were obtained by running the local perl scripts targetscan_60.pl and targetscan_61_context_scores.pl that were online available at the TargetScan website (http://www.targetscan.org/) [61]. Mature microRNA sequences were downloaded from miRBase release 18 [55]. To derive high efficacy targets, we filtered target predictions with a context score≤−0.1 [61].

To determine TF target genes, we downloaded the transcriptional start sites (TSSs) of all genes included in the mRNA expression data set from the hg19 assembly of the UCSC Table Browser [51] and the TSSs from our proliferation related microRNA genes from miRStart [62]. Further, we defined the promoter region to −/+ 2000 nucleotides around the TSSs. Genes having a TFBS that completely overlapped their promoter regions were considered as TF targets. We just considered human TFs that were expressed in at least one proliferation group of our OS cell samples (log2 intensity≥8).

### Enrichment of microRNA target genes

To assess the enrichment of microRNA target genes in the list of DE genes, a hypergeometric test was performed. Multiple test correction was done by determining the FDR according to Benjamini and Hochberg [58]. To account for the different number of target genes of the DE microRNAs, a permutation procedure was applied. We randomly selected the number of DE genes out of the genes in our mRNA expression data set and counted the number of random microRNA target genes. For each permutation an enrichment score (ES, -log10 p-value) was calculated. The permutation procedure was repeated 1,000 times. The resulting permutation p-values were obtained by counting the number of permuted ESs exceeding the observed one. This was done for all DE microRNAs separately.

### Fuzzy c-means clustering of DE microRNA targets

To classify DE microRNA targets according to their functional similarity, their GO semantic similarity scores based on biological process terms were computed using Resnik's information content approach of the GOSim package [63]. The resulting functional similarity scores for any target gene pair were listed in a similarity matrix, which was further utilized as distance matrix for clustering. We applied FCM [64] to classify microRNA target genes according to their functional similarity using the function fanny from the R cluster package [65]. The fuzziness parameter was estimated by Dunn's coefficient [66] among a range of 1.1 to 1.5 and the cluster number was estimated over a range of 2 to 15 using Dunn's index [67]. The Dunn coefficient (range 0–1) indicates the fuzziness of a cluster [66]. The Dunn index compared the between cluster variation to the within cluster variation and measures the cluster separation. A Dunn index >1 indicates a satisfying clustering [67].

The derived functional clusters were evaluated by running a GO enrichment analysis with the Bioconductor package GOStats [68]. Multiple test correction was performed by using Benjamini and Hochberg's FDR approach [58].

### Testing and evaluating microRNA and TF co-regulation

We tested for non-random microRNA and TF 3-node and 4-node motifs by using the hypergeometric test adapted from Sun *et al.* [14]. In contrast to them, we applied a different null model to derive p-values specific for the underlying microRNA and TF co-regulation pairs.

For the 3-node motifs we tested if a microRNA and TF pair had significantly more commonly DE target genes than computationally predicted target genes. In turn, co-regulation of microRNA and TF pairs in 4-node motifs was tested based on commonly regulated secondary target genes and compared to the genes with corresponding TFBS in the whole 1^st^-neighbor protein interaction network. The 1^st^-neighbor protein interaction network was determined by extracting all 1^st^-neighbors of microRNA target genes from the protein interaction data. Benjamini and Hochberg's FDR was used to adjust for multiple testing [58].

Furthermore, evaluation of significant pairs of microRNAs and TFs was performed by assessing the coexpression of genes targeted by the same microRNA and TF pair. The Pearson correlation was used as a measure for coexpression. Statistical significance was determined by a permutation procedure. We randomly chose the same number of genes targeted by 3-node and 4-node FFLs out of all genes annotated in the mRNA expression data and computed their correlation coefficients. The permutation procedure was repeated 1,000 times. Finally, we tested if the coexpression of the genes in the FFLs was significantly greater than in randomly selected gene pairs using the KS test.

### MicroRNA and TF co-regulatory network generation and analyses

The microRNA and TF co-regulatory networks were constructed by merging all 3-node and 4-node FFL motifs. The networks were modeled as graphs with nodes and edges. Nodes corresponded to microRNAs, TFs, or target genes and edges corresponded to microRNA-target regulation, TF-target regulation, or protein interactions. To identify crucial network players, we computed network centralities, namely node degree and betweenness, using the R package igraph [26]. The node degree is defined as the number of direct neighbors of a node in a network. Nodes having a high number of direct neighbors are thought to be important regulatory hubs inside the network. In contrast, a node's betweenness is a measure of the number of shortest paths between any pair of nodes that run through it [69].

To detect tightly connected groups of nodes in the network, we run the walktrap algorithm [70]. This algorithm finds modules in connected graphs. It is based on random walks and assumes that the random walker is trapped in dense parts of a network [26].

For further network evaluation we used the Functional Annotation Tool of the DAVID database [27]. The networks were visualized with Cytoscape 2.8.3 [71] and Cytoscape Web 1.0.2 [72].

## Supporting Information

Figure S1 **Enrichment of proliferation-related microRNAs.** The barplot of enrichment scores (ESs) of observed microRNA target genes (cyan) and randomly selected targets (grey). The ES of randomly selected microRNA targets is illustrated as mean±stdev. Per microRNA we computed 1,000 random ESs. P-values between observed and random ESs were obtained by counting the number of random ESs exceeding the observed one.
(PDF)

Figure S2 **Distribution of Dunn coefficients and indices determined by FCM clustering.** (**A**) Assessing the optimal fuzziness parameter. The plot illustrates the Dunn coefficients (y-axis) among a range of fuzziness parameters (x-axis) for different cluster numbers. The fuzziness was set to 1.1, where the Dunn coefficient distribution exceeds 0.5 for all cluster numbers. (**B**) Determining the optimal cluster number. The plot shows the Dunn indices (y-axis) among a range of cluster numbers (x-axis) for different fuzziness parameters. The optimal cluster number was set to 2, where the Dunn index reached its maximum value.
(PDF)

Figure S3 **Node degree distribution of the microRNA and TF co-regulatory networks.** The plots show the fraction of proteins (y-axis) among all node degrees (x-axis) from the microRNA and TF co-regulatory networks (grey) of (**A**) C1 and (**B**) C2. Different colors indicate distinct degree distributions of different node types. Horizontal lines mark the average node degree of individual node types. The values of the average node degrees are listed in the plots' legends.
(PDF)

Figure S4 **MicroRNA and TF co-regulatory network modules derived from C1.** The figure shows network modules defined by the walktrap algorithm. The modules C1.1 to C2.6 are labeled from (**A**) to (**F**). Node shapes correspond to the distinct node types: microRNAs (diamond), TFs (triangle), primary target (rectangle), and secondary target (ellipse). Yellow edges mark TF-DNA interactions, blue edges microRNA-target interactions, and dashed grey edges protein interactions. The red/green color code indicates the log2 FC.
(PDF)

Figure S5 **MicroRNA and TF co-regulatory network modules derived from C2.** The figure shows network modules defined by the walktrap algorithm. The modules C2.1 to C2.6 are labeled from (**A**) to (**F**). Node shapes correspond to the distinct node types: microRNAs (diamond), TFs (triangle), primary target (rectangle), and secondary target (ellipse). Yellow edges mark TF-DNA interactions, blue edges microRNA-target interactions, and dashed grey edges protein interactions. The red/green color code indicates the log2 FC. The C2.1 module implicated in negative regulation of differentiation of osteoblast cells is tagged with a red frame.
(PDF)

Figure S6 ***miR-9-5p*** **and** ***SP1*** **target gene associations.** The network is derived from the STRING 9.0 database [42]. It illustrates experimental and literature-mined functional associations between *miR-9-5p* and *SP1* target genes.
(TIF)

Figure S7 **Module C2.1 target gene associations.** The network is derived from the STRING 9.0 database [42]. It illustrates experimental and literature-mined functional associations between genes within the C2.1 network module.
(TIF)

Table S1 **Enrichment of proliferation-related microRNA target genes.** Results of the hypergeometric test to examine significantly enriched microRNA target genes within the list of DE genes. The table marks the total number of predicted target genes, the number of DE target genes, and the corresponding FDR.
(XLS)

Table S2 **Significant microRNA and TF co-regulatory 3-node motifs.** The table summarizes the number of common target genes of each non-random microRNA and TF co-regulatory pair with corresponding statistics.
(XLS)

Table S3 **Significant microRNA and TF co-regulatory 4-node motifs.** The table summarizes the number of interacting target genes of each non-random microRNA and TF co-regulatory pair with corresponding statistics.
(XLS)

Table S4 **Individual microRNA and TF co-regulatory 3-node and 4-node motifs of C1.**
(XLS)

Table S5 **Individual microRNA and TF co-regulatory 3-node and 4-node motifs of C2.**
(XLS)

Table S6 **MicroRNA and TF co-regulatory network hubs.** The table holds the node degree and betweenness parameters of hub genes within the co-regulatory network of C1 and C2 for each node type.
(XLS)

Table S7 **Over-represented KEGG pathways.**
(XLS)
