# Supplementary figures and images for: How MicroRNA and Transcription Factor Co-regulatory Networks Affect Osteosarcoma Cell Proliferation
Source: PLoS Comput Biol. 2013 Aug 29;9(8):e1003210. doi: 10.1371/journal.pcbi.1003210 (PMC3757060; doi:10.1371/journal.pcbi.1003210)

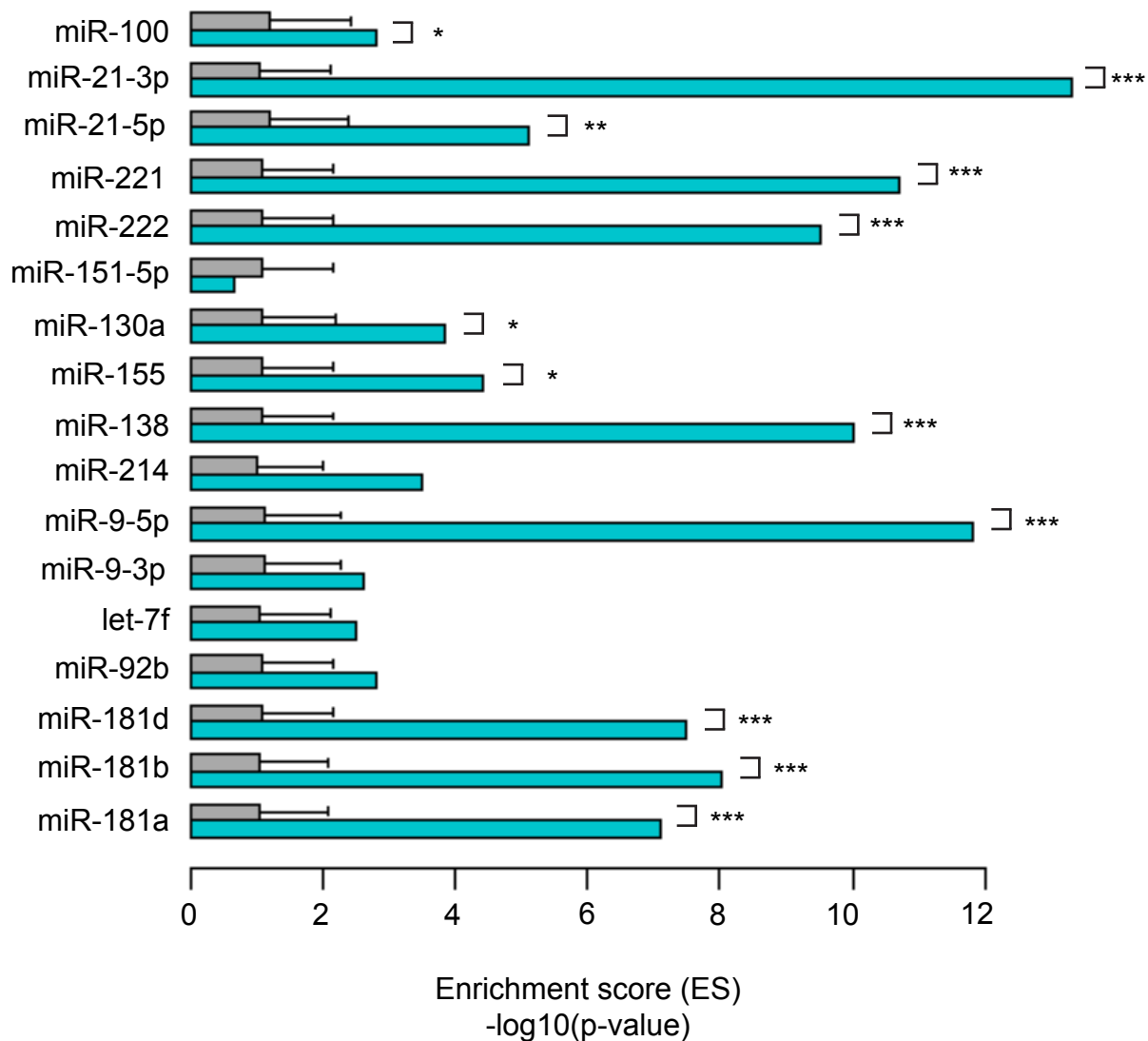

Supplement: Figure S1 — Enrichment of proliferation-related microRNAs. The barplot of enrichment scores (ESs) of observed microRNA target genes (cyan) and randomly selected targets (grey). The ES of randomly selected microRNA targets is illustrated as mean±stdev. Per microRNA we computed 1,000 random ESs. P-values between observed and random ESs were obtained by counting the number of random ESs exceeding the observed one. (PDF) [file pcbi.1003210.s001.pdf]

**A**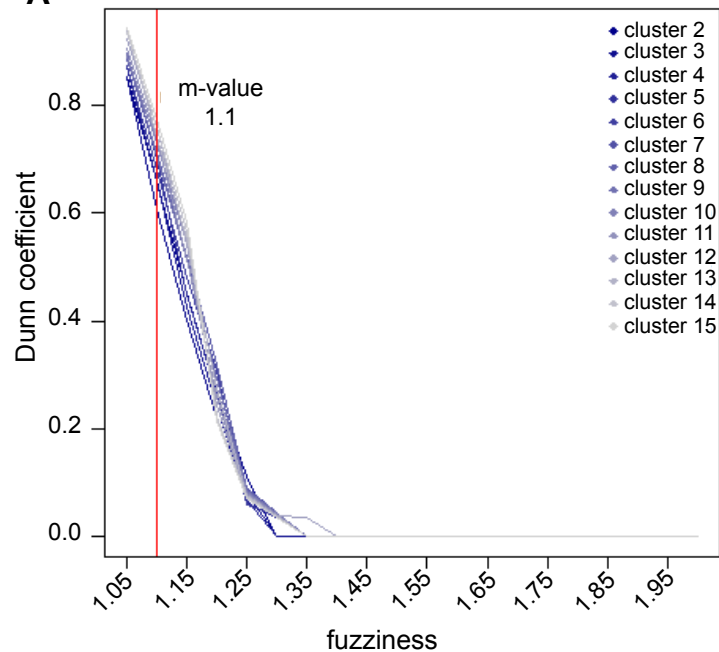**B**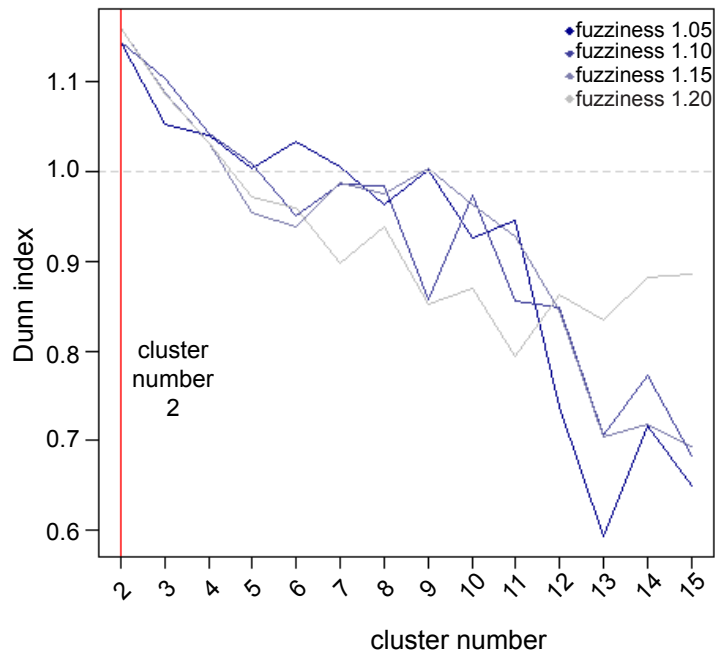

Supplement: Figure S2 — Distribution of Dunn coefficients and indices determined by FCM clustering. (A) Assessing the optimal fuzziness parameter. The plot illustrates the Dunn coefficients (y-axis) among a range of fuzziness parameters (x-axis) for different cluster numbers. The fuzziness was set to 1.1, where the Dunn coefficient distribution exceeds 0.5 for all cluster numbers. (B) Determining the optimal cluster number. The plot shows the Dunn indices (y-axis) among a range of cluster numbers (x-axis) for different fuzziness parameters. The optimal cluster number was set to 2, where the Dunn index reached its maximum value. (PDF) [file pcbi.1003210.s002.pdf]

**A**

C1 co-regulatory network

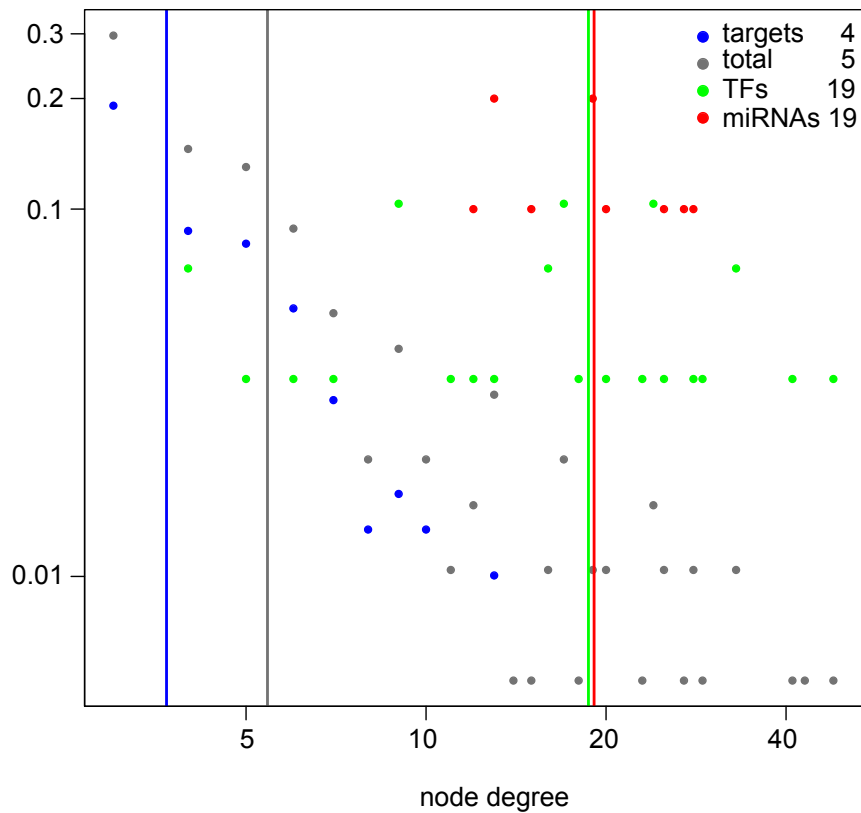**B**

C2 co-regulatory network

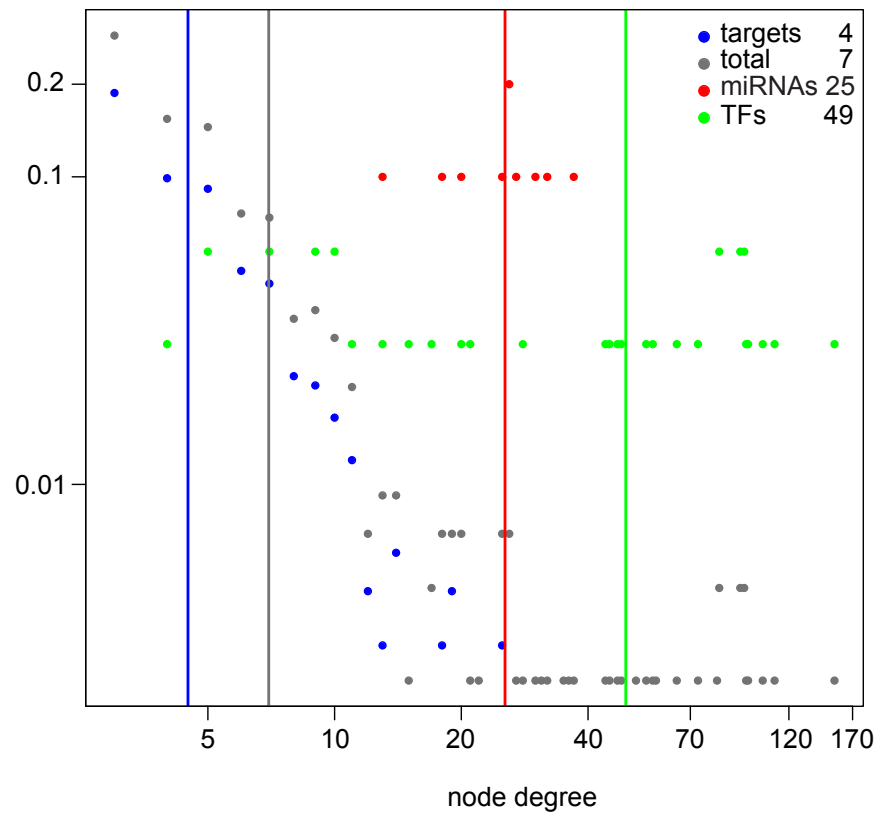

Supplement: Figure S3 — Node degree distribution of the microRNA and TF co-regulatory networks. The plots show the fraction of proteins (y-axis) among all node degrees (x-axis) from the microRNA and TF co-regulatory networks (grey) of (A) C1 and (B) C2. Different colors indicate distinct degree distributions of different node types. Horizontal lines mark the average node degree of individual node types. The values of the average node degrees are listed in the plots' legends. (PDF) [file pcbi.1003210.s003.pdf]

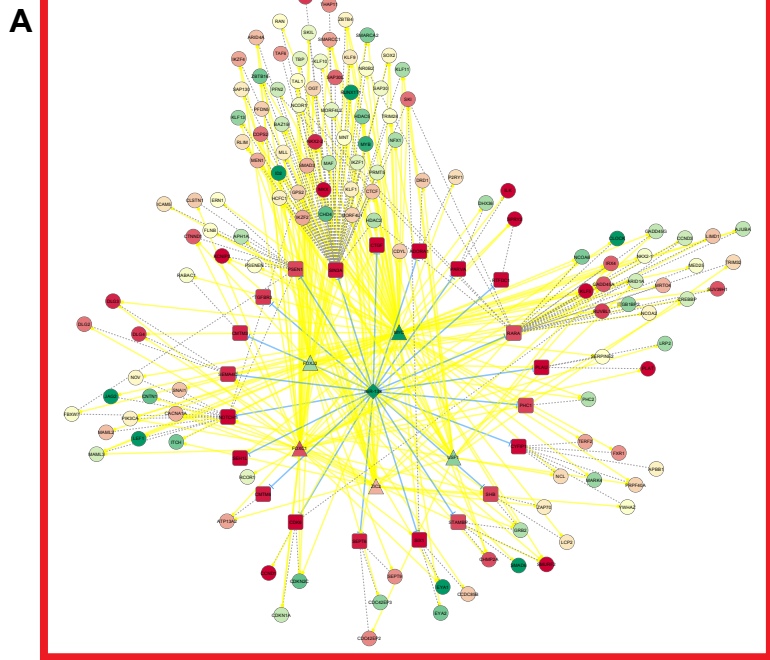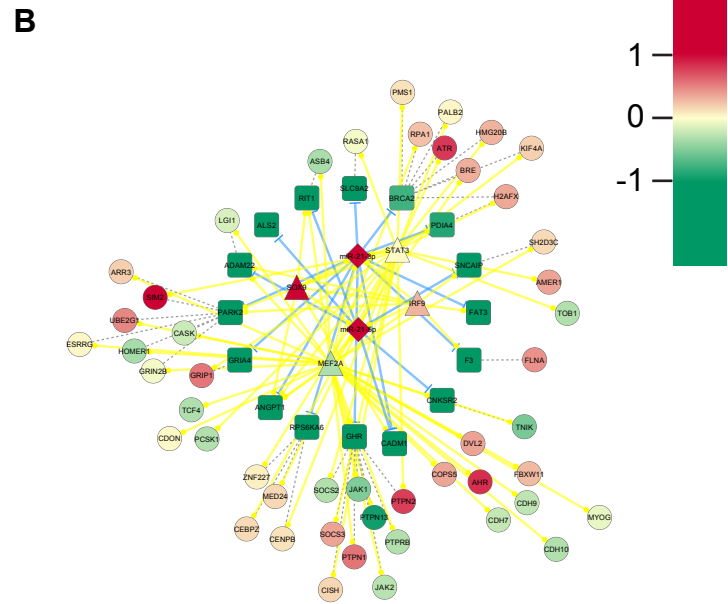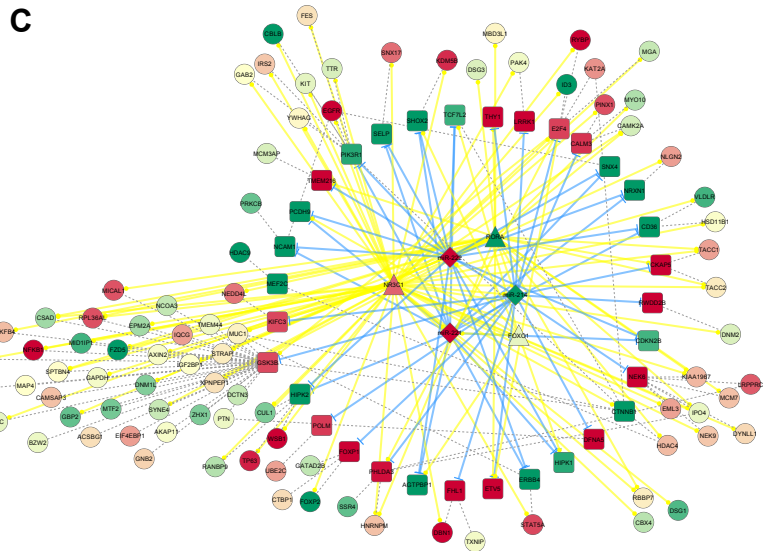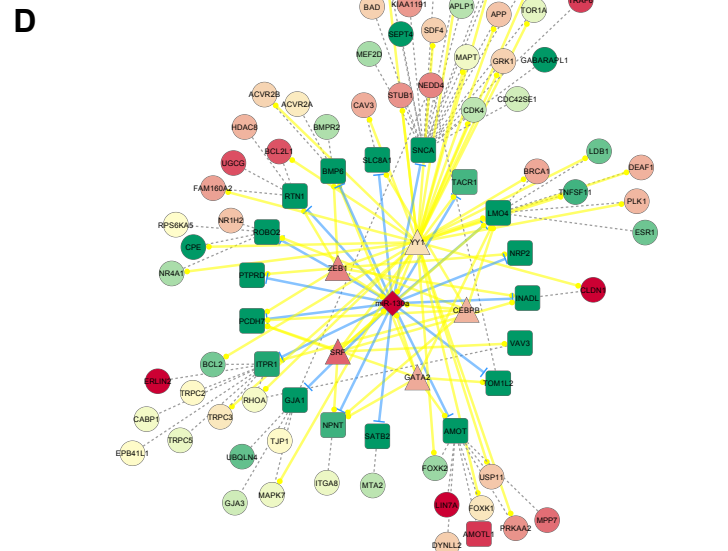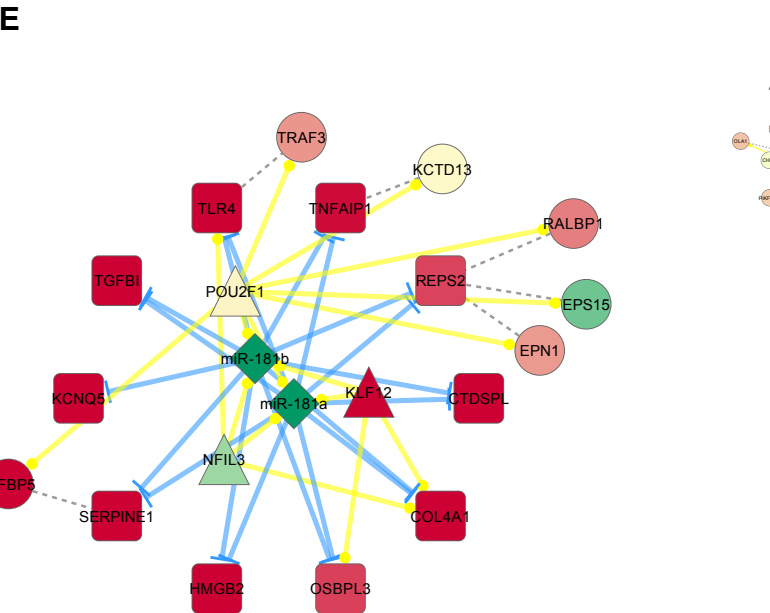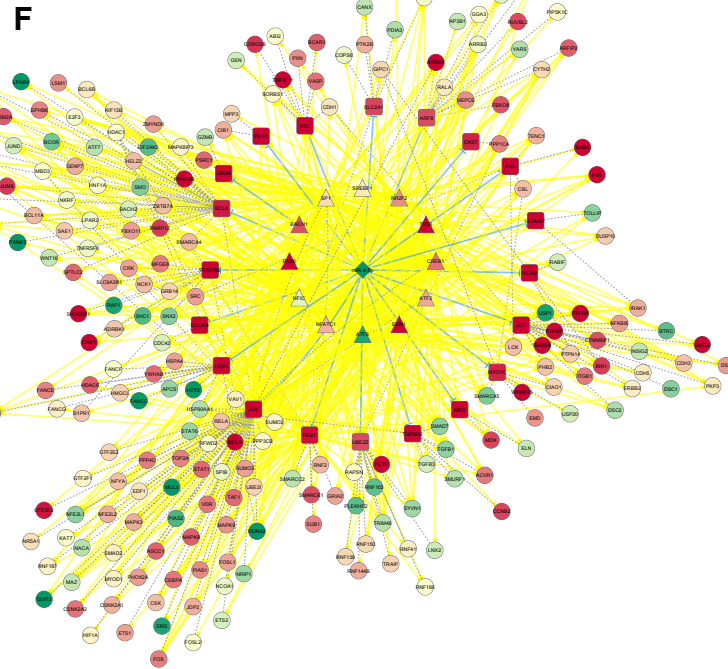

Supplement: Figure S5 — MicroRNA and TF co-regulatory network modules derived from C2. The figure shows network modules defined by the walktrap algorithm. The modules C2.1 to C2.6 are labeled from (A) to (F). Node shapes correspond to the distinct node types: microRNAs (diamond), TFs (triangle), primary target (rectangle), and secondary target (ellipse). Yellow edges mark TF-DNA interactions, blue edges microRNA-target interactions, and dashed grey edges protein interactions. The red/green color code indicates the log2 FC. The C2.1 module implicated in negative regulation of differentiation of osteoblast cells is tagged with a red frame. (PDF) [file pcbi.1003210.s005.pdf]

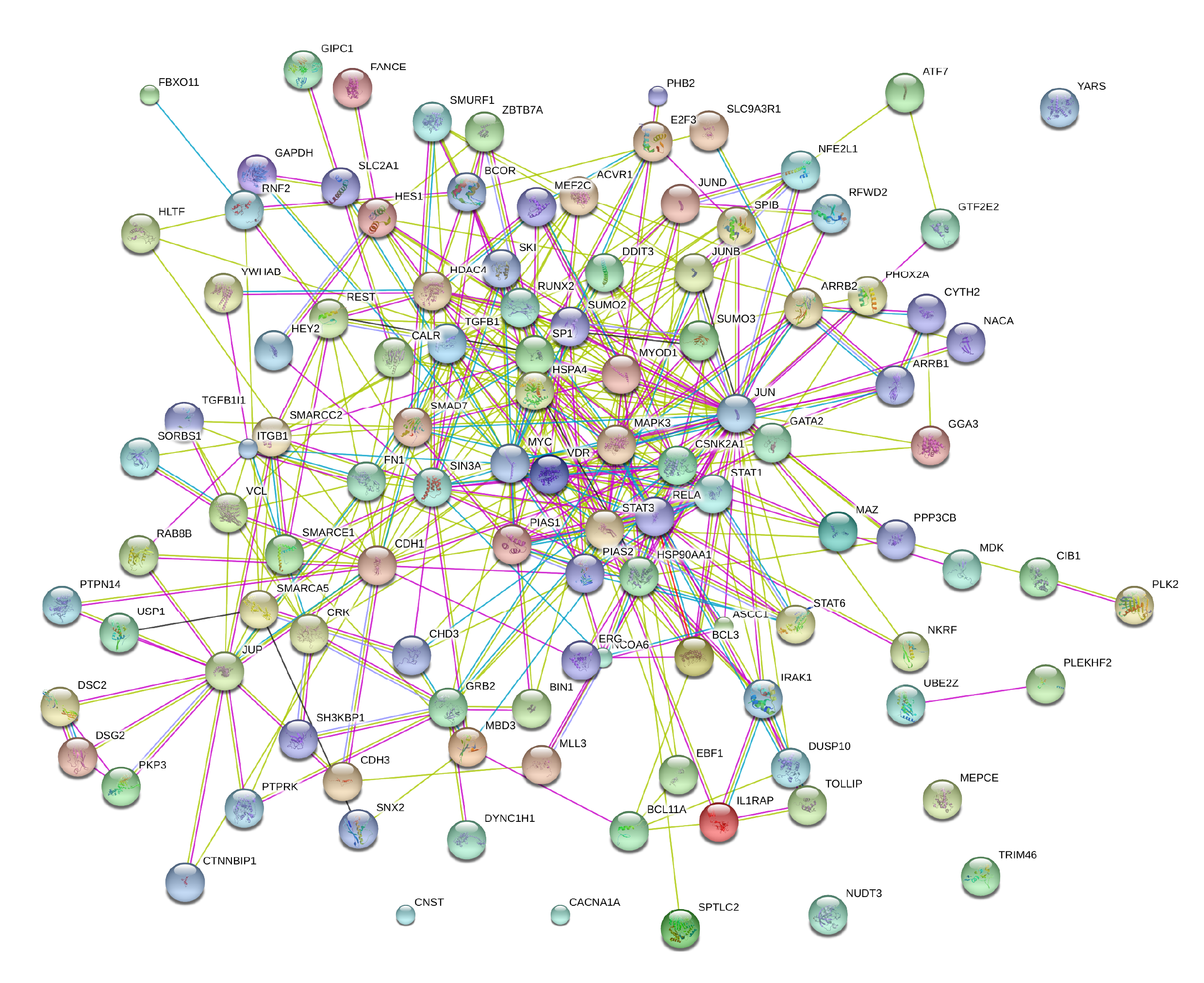

Supplement: Figure S6 — miR-9-5p and SP1 target gene associations. The network is derived from the STRING 9.0 database [42]. It illustrates experimental and literature-mined functional associations between miR-9-5p and SP1 target genes. (TIF) [file pcbi.1003210.s006.tif]

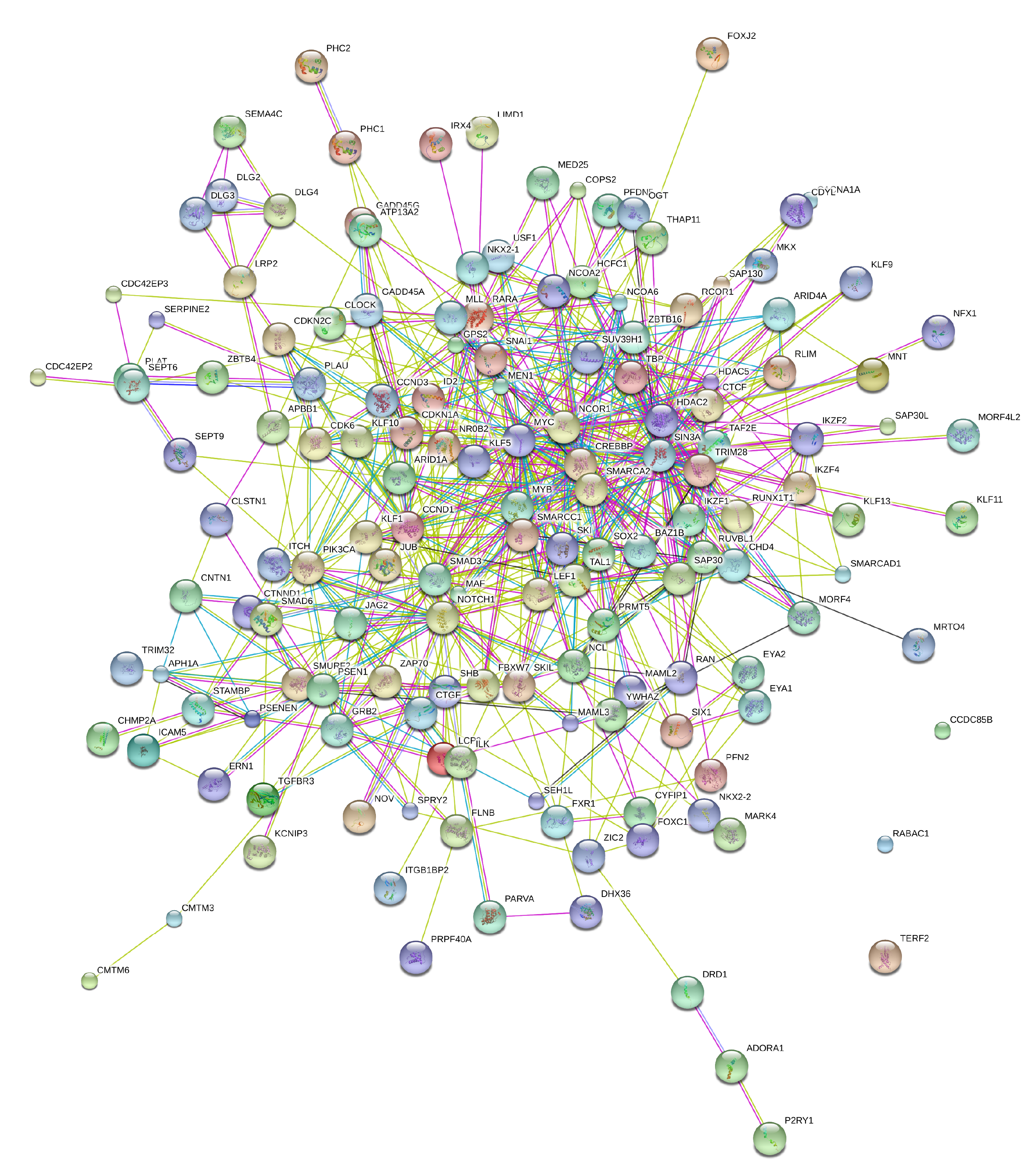

Supplement: Figure S7 — Module C2.1 target gene associations. The network is derived from the STRING 9.0 database [42]. It illustrates experimental and literature-mined functional associations between genes within the C2.1 network module. (TIF) [file pcbi.1003210.s007.tif]
