# Supplementary material for: How MicroRNA and Transcription Factor Co-regulatory Networks Affect Osteosarcoma Cell Proliferation
Source: PLoS Comput Biol. 2013 Aug 29;9(8):e1003210. doi: 10.1371/journal.pcbi.1003210 (PMC3757060; doi:10.1371/journal.pcbi.1003210)
Supplement: Figure S4 — MicroRNA and TF co-regulatory network modules derived from C1. The figure shows network modules defined by the walktrap algorithm. The modules C1.1 to C2.6 are labeled from (A) to (F). Node shapes correspond to the distinct node types: microRNAs (diamond), TFs (triangle), primary target (rectangle), and secondary target (ellipse). Yellow edges mark TF-DNA interactions, blue edges microRNA-target interactions, and dashed grey edges protein interactions. The red/green color code indicates the log2 FC. (PDF) [file pcbi.1003210.s004.pdf]

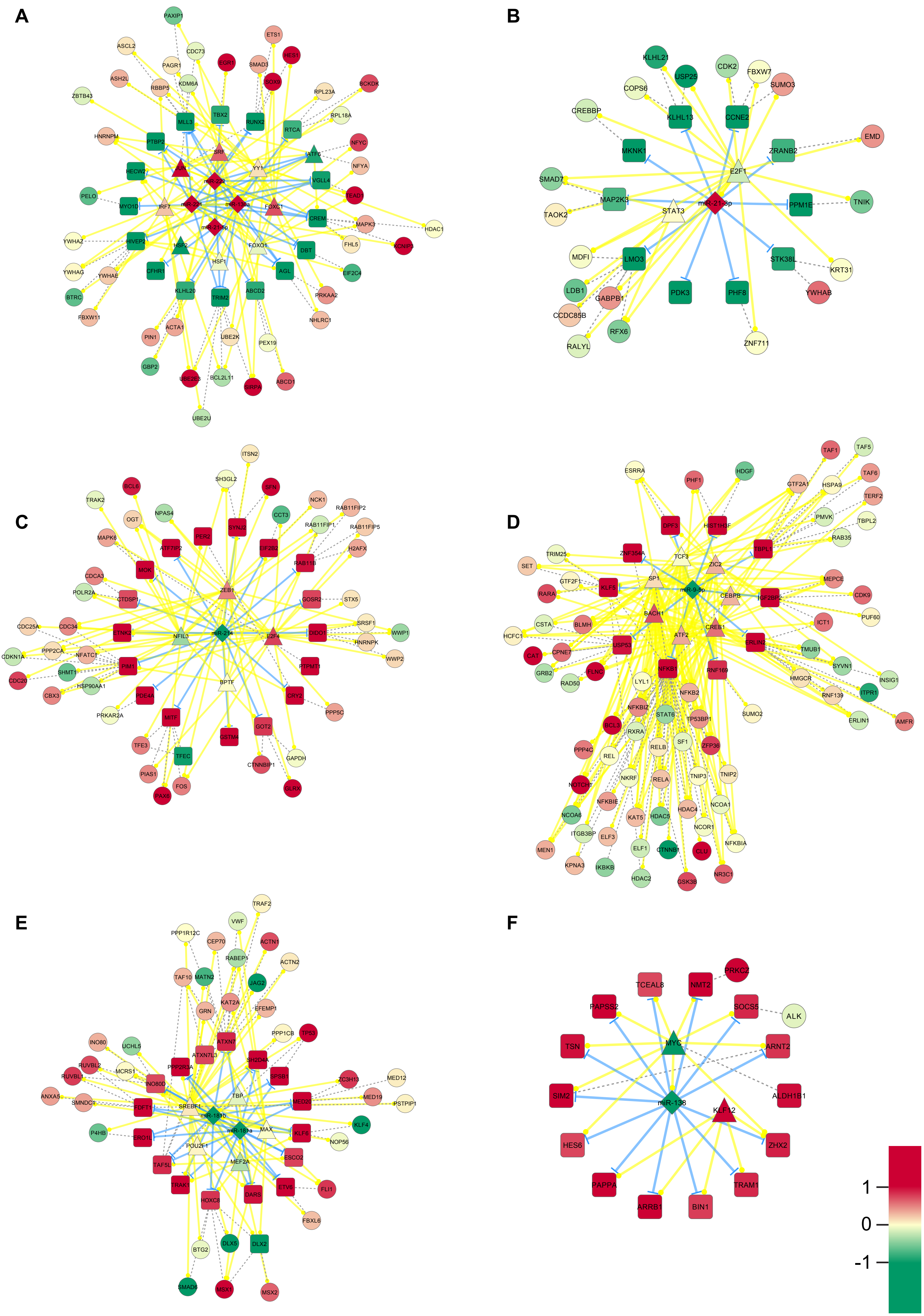
A
PAXIP1
ETS1
ASCL2
CDC73
HES1
PAGR1
EGR1
SMAD3
ASH2L
KDM6A
SOX9
BCKDK
RBBP5
RPL23A
ZBTB43
TBX2
RPL18A
MLL3
RUNX2
RTCA
HNRNPM
PTBP2
NFYC
SRF
ATF6
JUN
YY1
HECW2
NFYA
miR-222
VGLL4
PELO
TEAD1
MYO1D
IRF7
miR-221
miR-130a
FOXC1
CREM
MAPK3
HDAC1
miR-21-5p
YWHAZ
HIVEP2
HSF2
FOXO1
FHL5
KCNIP3
DBT
HSF1
YWHAG
YWHAE
CFHR1
AGL
EIF2C4
BTRC
KLHL20
ABCD2
TRIM2
PRKAA2
FBXW11
NHLRC1
ACTA1
PIN1
UBE2K
PEX19
GBP2
UBE2E3
BCL2L11
SIRPA
ABCD1
UBE2U
B
KLHL21
USP25
CDK2
FBXW7
COPS6
SUMO3
CREBBP
KLHL13
CCNE2
EMD
MKNK1
ZRANB2
E2F1
SMAD7
MAP2K3
miR-21-3p
PPM1E
TNIK
STAT3
TAOK2
MDFI
LMO3
STK38L
KRT31
LDB1
YWHAB
GABPB1
PDK3
PHF8
CCDC85B
RFX6
RALYL
ZNF711
C
ITSN2
SH3GL2
BCL6
SFN
TRAK2
NCK1
CCT3
RAB11FIP2
NPAS4
OGT
RAB11FIP1
RAB11FIP5
PER2
SYNJ2
MAPK6
ATF7IP2
EIF2B2
H2AFX
RAB11B
MOK
CDCA3
ZEB1
POLR2A
CTDSP1
GOSR2
STX5
SRSF1
CDC25A
CDC34
ETNK2
NFIL3
miR-214
E2F4
DIDO1
WWP1
HNRNPK
PPP2CA
NFATC1
CDKN1A
WWP2
PIM1
SHMT1
PTPMT1
CDC20
HSP90AA1
BPTF
CBX3
PDE4A
CRY2
PPP5C
PRKAR2A
MITF
TFE3
GOT2
GSTM4
TFEC
GAPDH
CTNNBIP1
PIAS1
FOS
PAX6
GLRX
D
TAF1
TAF5
ESRRA
HDGF
TAF6
PHF1
GTF2A1
HSPA9
TERF2
DPF3
HIST1H3F
PMVK
TBPL2
RAB35
TRIM25
ZNF354A
TCF3
TBPL1
SET
ZIC2
GTF2F1
SP1
MEPCE
RARA
KLF5
miR-9-5p
CEBPB
GTF2B2
CDK9
BACH1
PUF60
CSTA
BLMH
ICT1
HCFC1
ATF2
CREB1
USP53
ERLIN2
TMUB1
CAT
CPNE7
NFKB1
SYVN1
GRB2
FLNC
RNF169
HMGCR
INSIG1
RAD50
LYL1
ITPR1
NFKB2
RNF139
NFKBIZ
STAT6
TP53BP1
SUMO2
ERLIN1
AMFR
BCL3
RXRA
SF1
PPP4C
RELB
ZFP36
REL
TNIP3
TNIP2
NOTCH1
NKRF
RELA
NFKBIE
HDAC4
NCOA1
NCOA6
KAT5
HDAC5
NCOR1
ITGB3BP
ELF3
NFKBIA
MEN1
ELF1
CTNNB1
CLU
KPNA3
IKBKB
HDAC2
GSK3B
NR3C1
E
TRAF2
VWF
PPP1R12C
CEP70
ACTN1
RABEP1
ACTN2
TAF10
MATN2
JAG2
KAT2A
EFEMP1
GRN
ATXN7
PPP1CB
TP53
INO80
UCHL5
ATXN7L3
SH2D4A
RUVBL2
MCRS1
PPP2R3A
RUVBL1
INO80D
SPSB1
ZC3H13
MED12
TBP
ANXA5
SMNDC1
SREBF1
MED26
MED19
PSTPIP1
FDFT1
miR-181b
miR-181a
KLF4
P4HB
ERO1L
MAX
KLF6
NOP56
POU2F1
TAF5L
MEF2A
ESCO2
TRAK1
ETV6
FLI1
HOXC8
DARS
FBXL6
DLX5
DLX2
BTG2
SMAD6
MSX1
MSX2
F
PRKCZ
TCEAL8
NMT2
PAPSS2
SOCS5
ALK
TSN
MYC
ARNT2
SIM2
ALDH1B1
miR-138
KLF12
HES6
ZHX2
PAPPA
TRAM1
ARRB1
BIN1
1
0
-1
